# Beneficial effects of colchicine for moderate to severe COVID-19: a randomised, double-blinded, placebo-controlled clinical trial

**DOI:** 10.1136/rmdopen-2020-001455

**Published:** 2021-02-04

**Authors:** Maria Isabel Lopes, Leticia P Bonjorno, Marcela C Giannini, Natalia B Amaral, Pamella Indira Menezes, Saulo Musse Dib, Samara Libich Gigante, Maira N Benatti, Uebe C Rezek, Laerte L Emrich-Filho, Betania A A Sousa, Sergio C L Almeida, Rodrigo Luppino Assad, Flavio P Veras, Ayda Schneider, Tamara S Rodrigues, Luiz O S Leiria, Larissa D Cunha, Jose C Alves-Filho, Thiago M Cunha, Eurico Arruda, Carlos H Miranda, Antonio Pazin-Filho, Maria Auxiliadora-Martins, Marcos C Borges, Benedito A L Fonseca, Valdes R Bollela, Cristina M Del-Ben, Fernando Q Cunha, Dario S Zamboni, Rodrigo C Santana, Fernando C Vilar, Paulo Louzada-Junior, Rene D R Oliveira

**Affiliations:** 1 Department of Internal Medicine, Ribeirao Preto Medical School, University of Sao Paulo, Ribeirao Preto, Brazil; 2 Department of Pharmacology, Ribeirao Preto Medical School, University of Sao Paulo, Ribeirao Preto, Brazil; 3 Department of Cell Biology, Ribeirao Preto Medical School, University of Sao Paulo, Ribeirao Preto, Brazil; 4 Department of Emergency Medicine, Ribeirao Preto Medical School, University of Sao Paulo, Ribeirao Preto, Brazil; 5 Department of Surgery and Anatomy, Ribeirao Preto Medical School, University of Sao Paulo, Ribeirao Preto, Brazil; 6 Department of Neuroscience and Behaviour, Ribeirao Preto Medical School, University of Sao Paulo, Ribeirao Preto, Brazil

**Keywords:** inflammation, cytokines, outcome assessment, health care

## Abstract

**Objective:**

To evaluate whether the addition of colchicine to standard treatment for COVID-19 results in better outcomes.

**Design:**

We present the results of a randomised, double-blinded, placebo-controlled clinical trial of colchicine for the treatment of moderate to severe COVID-19, with 75 patients allocated 1:1 from 11 April to 30 August 2020. Colchicine regimen was 0.5 mg thrice daily for 5 days, then 0.5 mg twice daily for 5 days. The primary endpoints were the need for supplemental oxygen, time of hospitalisation, need for admission and length of stay in intensive care unit and death rate.

**Results:**

Seventy-two patients (36 for placebo and 36 for colchicine) completed the study. Median (and IQR) time of need for supplemental oxygen was 4.0 (2.0–6.0) days for the colchicine group and 6.5 (4.0–9.0) days for the placebo group (p<0.001). Median (IQR) time of hospitalisation was 7.0 (5.0–9.0) days for the colchicine group and 9.0 (7.0–12.0) days for the placebo group (p=0.003). At day 2, 67% versus 86% of patients maintained the need for supplemental oxygen, while at day 7, the values were 9% versus 42%, in the colchicine and the placebo groups, respectively (log rank; p=0.001). Two patients died, both in placebo group. Diarrhoea was more frequent in the colchicine group (p=0.26).

**Conclusion:**

Colchicine reduced the length of both, supplemental oxygen therapy and hospitalisation. The drug was safe and well tolerated. Once death was an uncommon event, it is not possible to ensure that colchicine reduced mortality of COVID-19.

**Trial registration number:**

RBR-8jyhxh.

**Key messages**

**What is already known about this subject?**

- Systemic inflammation is the hallmark of hospitalised patients due to COVID-19, for which there is no specific treatment but supportive care and attempts to control the immune activation.
- By diminishing the activation of leucocytes, colchicine may be an intervention worthy of being tested.

**What does this study add?**

- This work presents data of a randomised controlled trial (RCT) of colchicine for hospitalised patients with COVID-19: the drug reduced the length of the need for supplemental oxygen and the length of hospitalisation.
- Colchicine was safe and well tolerated. The most common adverse event was diarrhoea. Arrhythmia was absent.

**How might this impact on clinical practice??**

- This is the first RCT on colchicine for COVID-19. Colchicine may be considered as an adjunctive therapy for hospitalised patients with moderate to severe COVID-19.

## Introduction

Systemic inflammation is the hallmark of moderate to severe cases of COVID-19.^1^ Its outbreak has already sent millions to infirmaries and intensive care units (ICUs) throughout the world, mainly due to pulmonary infiltrates resulting in the severe acute respiratory syndrome (SARS).^2^ High levels of interleukin (IL)-1β, IL-6, IL-18 and tumour necrosis factor (TNF) are some of the many immunological disturbances in the pathophysiology of the high inflammatory status of COVID-19,^3^ which counts, moreover, with markedly elevation of serum C reactive protein (CRP) and uncommon neutrophilia and lymphopaenia.^4 5^ Neutrophils release neutrophil extracellular traps (NETs), which were found to be toxic to lung epithelial cells in vitro. Furthermore, high levels of NETs were present in the plasma of patients with COVID-19 compared with healthy controls, and the presence of these cellular components was at least 10 times higher in tracheal aspirates than in plasma of the same patients, raising the question whether they have a role in the lung lesions.^6^

The inflammasome of NOD-, LRR- and pyrin domain-containing protein 3 (NLRP3) may be important in certain antiviral responses.^7^ After viral activation of the protein complex, mainly in monocytes and antigen-presenting tissue cells, its constituent pro-caspase-1 suffers autocleavage and, by its turn, cleaves pro-IL-1β and pro-IL-18 to their active form: IL-1β and IL-18.^8^ Both products activate B, T and NK cells in addition to stimulating the release of other inflammatory cytokines.^9^ It seems appropriate to infer that an aberrant activation of inflammasome underlies the ‘hyper’ inflammation found in hospitalised patients with COVID-19.

For decades, colchicine has been successfully used for the treatment and prevention of crystal-induced arthritis, for example, gout. Systemic autoinflammatory diseases such as familial Mediterranean fever and Behçet’s disease are conditions in which colchicine use may be necessary continuously.^10^ Much of this success comes from its direct effect on phagocytes residing or migrating into the synovial joints, vessel walls or other tissues, leading to inflammasome inhibition and impaired production and release of IL-1β^11^ and NETs.^12^ In all of these situations, the drug is well tolerated, and its adverse effects are broadly recognised.

Piantoni *et al*^13^ discussed the rationale of its use for the treatment of COVID-19, with focus on the control of the systemic inflammation caused by SARS-CoV-2 infection. A case–control study^14^ and an open-label clinical trial^15^ of colchicine for COVID-19 have results published hitherto.

## Methods

### Trial design

We conducted a randomised, double-blinded, placebo-controlled clinical trial to evaluate the use of colchicine for the treatment of hospitalised patients with moderate to severe COVID-19. The randomisation was performed 1:1 for placebo or colchicine by using the online tool at https://www.randomizer.org/.

To estimate the sample size, we took into account the parallelism of the groups and the 1:1 randomisation, with a minimal difference of effect of 0.30 attributed to the intervention on the primary endpoints. Alpha and beta errors of, respectively, 0.05 and 0.10, were predetermined, which resulted in a minimal number of 27 participants per group. Considering the prominent need of efficient therapies for COVID-19, besides limitations to conduct a clinical trial in a single centre, a minimal number of 60 patients seemed to be appropriate whether randomised into two groups of 30 patients.

The trial is registered on the National Registry under the alphanumeric code RBR-8jyhxh (http://www.ensaiosclinicos.gov.br/rg/RBR-8jyhxh/). All patients signed the consent form.

### Intervention

Patients of the intervention arm received colchicine 0.5 mg thrice daily for 5 days, then 0.5 mg twice daily for 5 days; if body weight ≥80 kg, the first dose was 1.0 mg. Whether a patient had chronic kidney disease, with glomerular filtration rate under 30 mL/min/1.73 m2, colchicine dose was reduced to 0.25 mg thrice daily for 5 days, then 0.25 mg twice daily for 5 days, no matter the body weight.

All participants received the institutional treatment for COVID-19 with azithromycin 500 mg once daily for up to 7 days, hydroxychloroquine 400 mg twice daily for 2 days, then 400 mg once daily for up to 8 days and unfractionated heparin 5000 UI thrice daily until the end of hospitalisation. During the study conduction, the first results of the RECOVERY Collaborative Group were launched,^16^ showing the benefits of adding a glucocorticoid to the treatment of COVID-19. Then, methylprednisolone 0.5 mg/kg/day for 5 days was added to the institutional protocol, for use if the patient’s need for supplemental oxygen was equal to 6 L/min or more. Study and institutional protocol drugs were suspended when participants reached good clinical and laboratory parameters and could be discharged.

### Study population

For a matter of selection criteria, we classified patients according to the severity of COVID-19: the mild form of the disease was defined in patients with flu-like symptoms without dyspnoea and imaging findings of pneumonia; the moderate form was defined in patients with fever, dyspnoea and imaging findings of pneumonia; the severe form in those with the same findings of moderate form plus respiratory rate ≥30 times per minute or oxygen saturation (SatO_2_) ≤92%; and the critical form was defined when patients presented respiratory failure or shock.^17 18^ The inclusion criteria were: individuals hospitalised with moderate or severe forms of COVID-19 diagnosed by RT-PCR in nasopharyngeal swab specimens and lung CT scan involvement compatible with COVID-19 pneumonia; older than 18 years; body weight >50 kg; normal levels of serum Ca^2+^ and K^+^; QT interval <450 ms at 12 derivations ECG (according to the Bazett formula) and negative serum or urinary β-HCG if woman under 50 years. The exclusion criteria were defined as: mild form of COVID-19 or in need for ICU admission; diarrhoea resulting in dehydration; known allergy to colchicine; diagnosis of porphyria, myasthenia gravis or uncontrolled arrhythmia at enrolment; pregnancy or lactation; metastatic cancer or immunosuppressive chemotherapy; regular use of digoxin, amiodarone, verapamil or protease inhibitors; chronic liver disease with hepatic failure; and inability to understand the consent Form.

### Setting

Patients were evaluated daily, and blood collection for general laboratory tests were performed at days 0, 2, 4 and 7 if discharge did not happen before. Twelve derivations ECG were performed each 24–48 hours. The definition of requirement of oxygen supply was a measure of SatO_2_ ≤92% at rest. The criteria for discharging patients from the hospital were the absence of dyspnoea and SatO_2_ >92%, both for at least 48 consecutive hours.

### Main endpoints

The primary endpoints were clinical parameters, such as the time of need for supplemental oxygen; time of hospitalisation; need for admission and length of stay in ICU; and death rate and causes of mortality. As secondary endpoints, we assessed clinical and laboratory parameters: measures of serum CRP, serum lactate dehydrogenase (LDH) and relation neutrophil to lymphocyte of peripheral blood samples from day 0 to day 7; the number, type and severity of adverse events; frequency of interruption of the study protocol due to adverse events; and frequency of QT interval above 450 ms.

### Statistical analysis

We present descriptive statistics as absolute numbers and percentage or median and IQR. Absolute numbers and percentage were compared with Fisher’s exact test. Comparisons of clinical and laboratory parameters expressed in median and IQR were done through Mann-Whitney test. Additionally, Kaplan-Meier survival curves were performed, with analysis by Mantel-Haenszel log rank test, to compare the time to abandon supplemental oxygen and time to discharge between the groups. Kruskall-Wallis test was used for comparisons of laboratory exams at the four blood collection times, followed by Dunn’s Multiple Comparison test. For all tests, p<0.05 was considered for statistical significance.

## Results

### Participants

**Figure 1:**
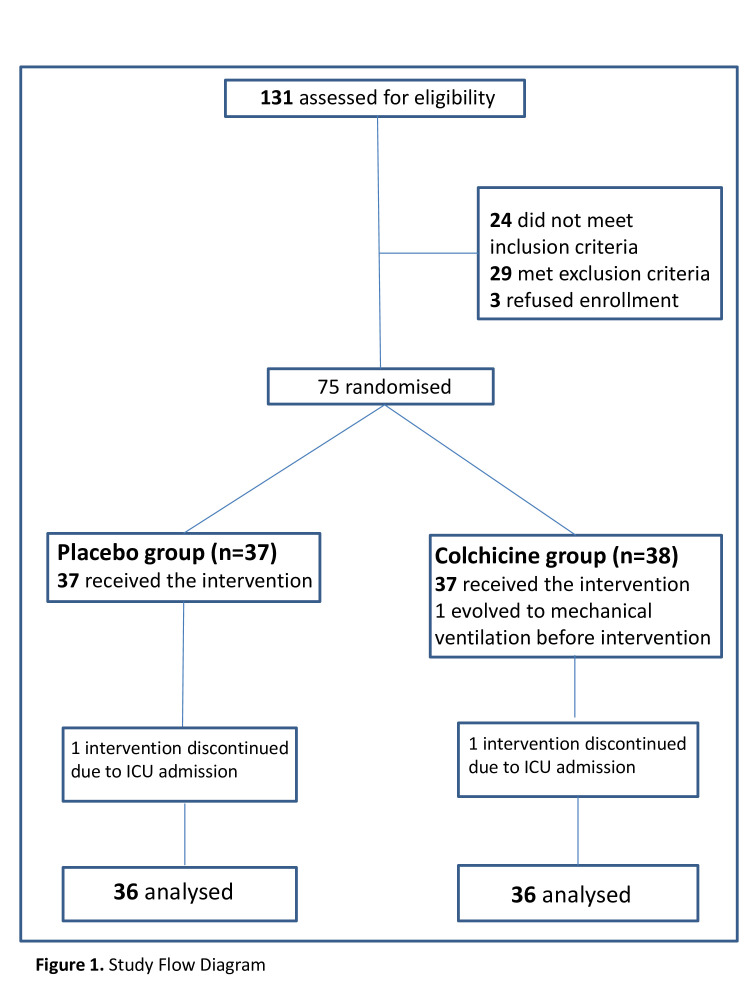
Study flow diagram. ICU, intensive care unit.

The enrolment started on 11 April and stopped on 31 August 2020. We assessed 131 patients and included 75 for randomisation as shown in figure 1. The baseline laboratory and clinical characteristics of the 72 patients who completed the study are presented in table 1. All patients received the institutional protocol treatment with hydroxychloroquine, azithromycin and heparin. Twenty-five and 24 patients, respectively, for colchicine and placebo groups received methylprednisolone. No treatment, institutional or interventional, was interrupted due to adverse events. The groups were similar in terms of demographic characteristics, clinical status and laboratory evaluation at baseline. There was a slight predominance of men in the colchicine group. Moreover, 35 out of 36 patients in the colchicine group had body mass index (BMI) above 25.0 kg/m^2^ (data not shown) and the group median arterial oxygen partial pressure (PaO_2_)/fractional inspired oxygen (FiO_2_) was 50.0 lower, for both parameters with no statistical difference.

**Table 1:** COVID-19 patients characteristics at baseline

	Placebo group (n=36)	Colchicine group (n=36)	P value
Demographics			
Men (n (%))	14 (39)	19 (53)	0.34
Age (years; median (IQR))	55.0 (42.0–67.0)	54.5 (42.5–64.5)	0.93
Time of symptoms (days; median (IQR))	8.0 (7.0–11.0)	9.5 (7.5–11.0)	0.15
Comorbidities (n (%))			
Smoking currently or formerly	9 (25)	7 (19)	0.78
Respiratory diseases	5 (14)	4 (11)	1
Cardiovascular diseases	16 (44)	17 (47)	1
Diabetes mellitus	15 (42)	13 (36)	0.81
Dyslipidaemia	12 (33)	10 (28)	0.80
BMI (kg/m^2^; median (IQR))	29.7 (26.3–36.0)	33.5 (28.6–37.8)	0.17
Clinical picture of COVID-19 (n (%))			
Fever	33 (92)	32 (89)	1
Cough	36 (100)	36 (100)	1
Fatigue	15 (42)	19 (53)	0.48
Myalgia	22 (61)	19 (53)	0.63
Diarrhoea	8 (22)	11 (31)	0.59
Without supplemental oxygen	4 (11)	1 (3)	0.36
On oxygen support (n (%)), (L/min; median (IQR))	24 (67), (3; 2.0–3.5)	28 (78), (3; 2.0–4.0)	0.65
On high flow	8 (22)	7 (19)	1
Mechanical ventilation	0	0	1
PaO_2_/FiO_2_ at enrolment (median (IQR))	278 (197–313)	228 (196–272)	0.25
qSOFA ≥1	34 (94)	34 (94)	1
SOFA	2.5 (1–3)	2.0 (1–3)	0.80
Laboratory findings			
Haemoglobin(g/dL; median (IQR))	12.9 (11.8–14.2)	13.3 (12.5–14.6)	0.44
Neutrophils(/mm^3^; median (IQR))	5450 (3800–7300)	5750 (3500–7650)	0.98
Lymphocytes(/mm^3^; median (IQR))	1300 (900–2050)	1100 (900–1550)	0.16
Neutrophil/Lymphocyte(median (IQR))	3.34 (2.46–6.00)	3.86 (2.80–7.44)	0.40
Platelets(/mm^3^; median (IQR))	2 40 000 (1 70 000–2 88 000)	2 26 000 (2 01 000–3 18 000)	0.79
Creatinine(mg/dL; median (IQR))	0.81 (0.64–1.05)	0.90 (0.70–1.01)	0.51
C-reactive protein(mg/dL; median (IQR))	9.3 (5.8–15.1)	9.2 (6.6–12.6)	0.91
Lactate dehydrogenase(U/L; median (IQR))	374 (301–477)	351 (299–473)	0.76
D-dimer(mcg/mL; median (IQR))	1.12 (0.63–1.77)	1.40 (0.92–1.92)	0.12
Ferritin(ng/mL; median (IQR))	597 (285–1289)	723 (436–1173)	0.43
Aspartate aminotransferase(U/L; median (IQR))	37 (27–63)	44 (28–70)	0.43
Alanine aminotransferase(U/L; median (IQR))	35 (24–48)	45 (33–80)	0.18
Medications			
Hydroxychloroquine(n (%))	36 (100)	36 (100)	1
Azithromycin(n (%))	36 (100)	36 (100)	1
Unfracionated heparin(n (%))	36 (100)	36 (100)	1
Methylprednisolone(n (%))	24 (67)	25 (69)	1

BMI, body mass index; D-dimer, dimerised plasma fragment D; FiO_2_, fractional inspired oxygen; PaO_2_, Arterial oxygen partial pressure; qSOFA, quick SOFA; SOFA, Sequential Organ Failure Assessment Score.

### Outcomes

At least half of the patients receiving colchicine stopped using supplemental oxygen for the fourth day (median 4.0; IQR 2.0–6.0 days) of intervention, while the same happened to the patients receiving placebo on the seventh day (median 6.5; IQR 4.0–9.0 days; p<0.001). Significant difference (p=0.003) between the groups was found for the time of hospitalisation, in detriment of the placebo group (median 7.0; IQR 5.0–9.0 days vs median 9.0; IQR 7.0–12.0 days).

Two and four patients, respectively, for colchicine and placebo groups needed admission to ICU. The ICU assistant team preferred to stop the study intervention for one patient per group. For this reason, these two patients were taken off the final analysis. Although the interventions were stopped, both patients were followed for some main outcomes: the time of treatment before ICU admission was 2 and 3 days, the length of stay in ICU was 12 and 11 days and the time of hospitalisation was 23 and 26 days, respectively, for colchicine and placebo groups. Two patients of the placebo group died (two male; death rate of 6%) and none of the colchicine group. The cause of death was ventilator-associated pneumonia in both cases. No statistical analysis was performed for the need for admission in ICU and the death rate due to the small number of events for each group.

**Figure 2:**
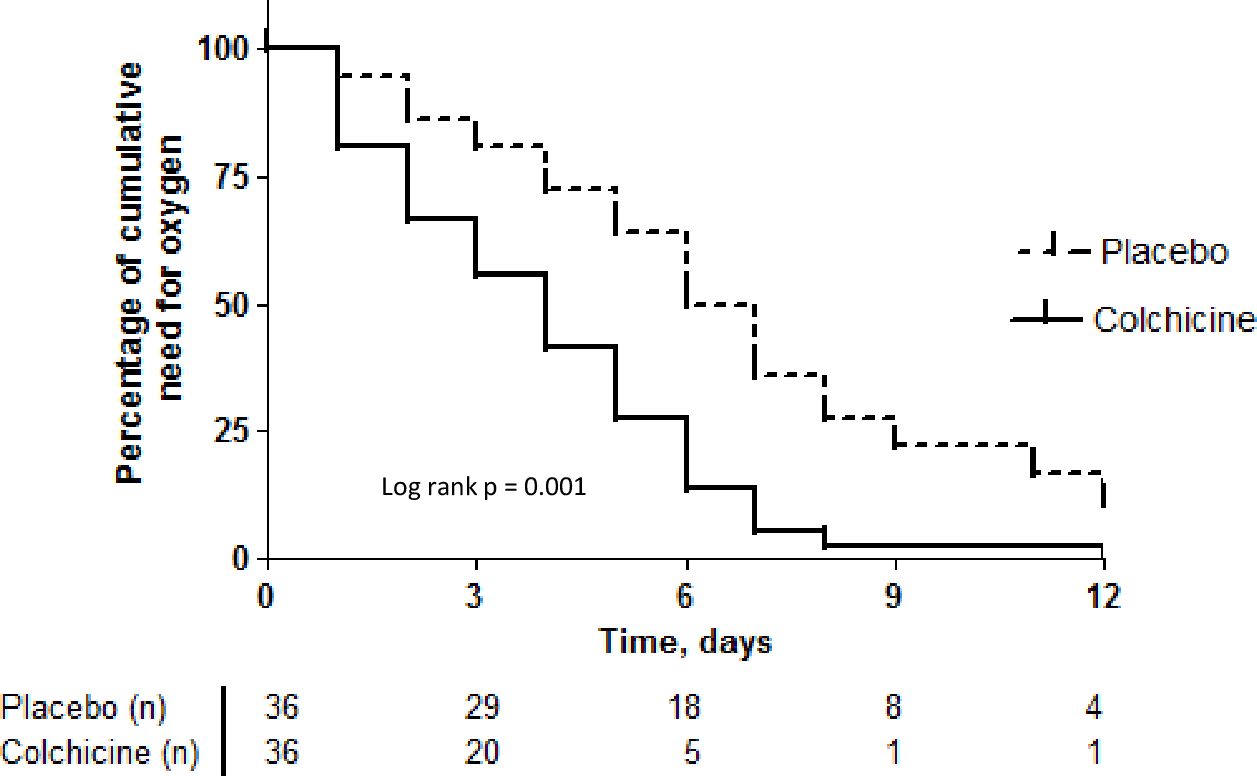
Kaplan-Meier curves of time to the end of need for supplemental oxygen for both groups.

**Figure 3:**
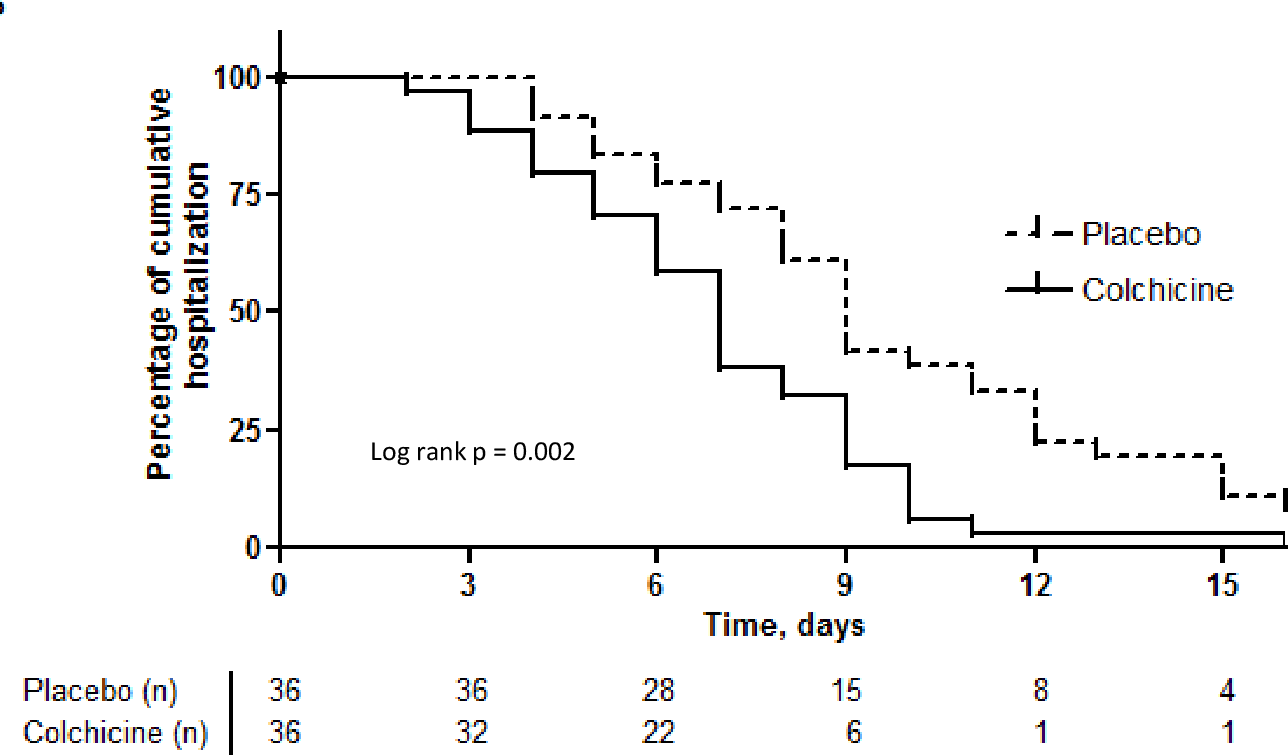
Kaplan-Meier curves of time to clinical improvement and discharge for both groups.

Kaplan-Meier survival curves for the need for supplemental oxygen and the maintenance of hospitalisation are depicted in figures 2 and 3, respectively. At day 2, 67% versus 86% of patients maintained the need for supplemental oxygen, while at day 7, these values were 9% versus 42%, in the colchicine and placebo groups, respectively (log rank test, 10.6; p=0.001). Hospitalisation was maintained for 42% versus 72% of patients at day 7 and 9% versus 39% at day 10, in the colchicine and placebo groups, respectively (log rank test, 9.2; p=0.002). Both outcomes presented similar behaviour, once the last is at large extension a consequence of the first one.

**Figure 4:**
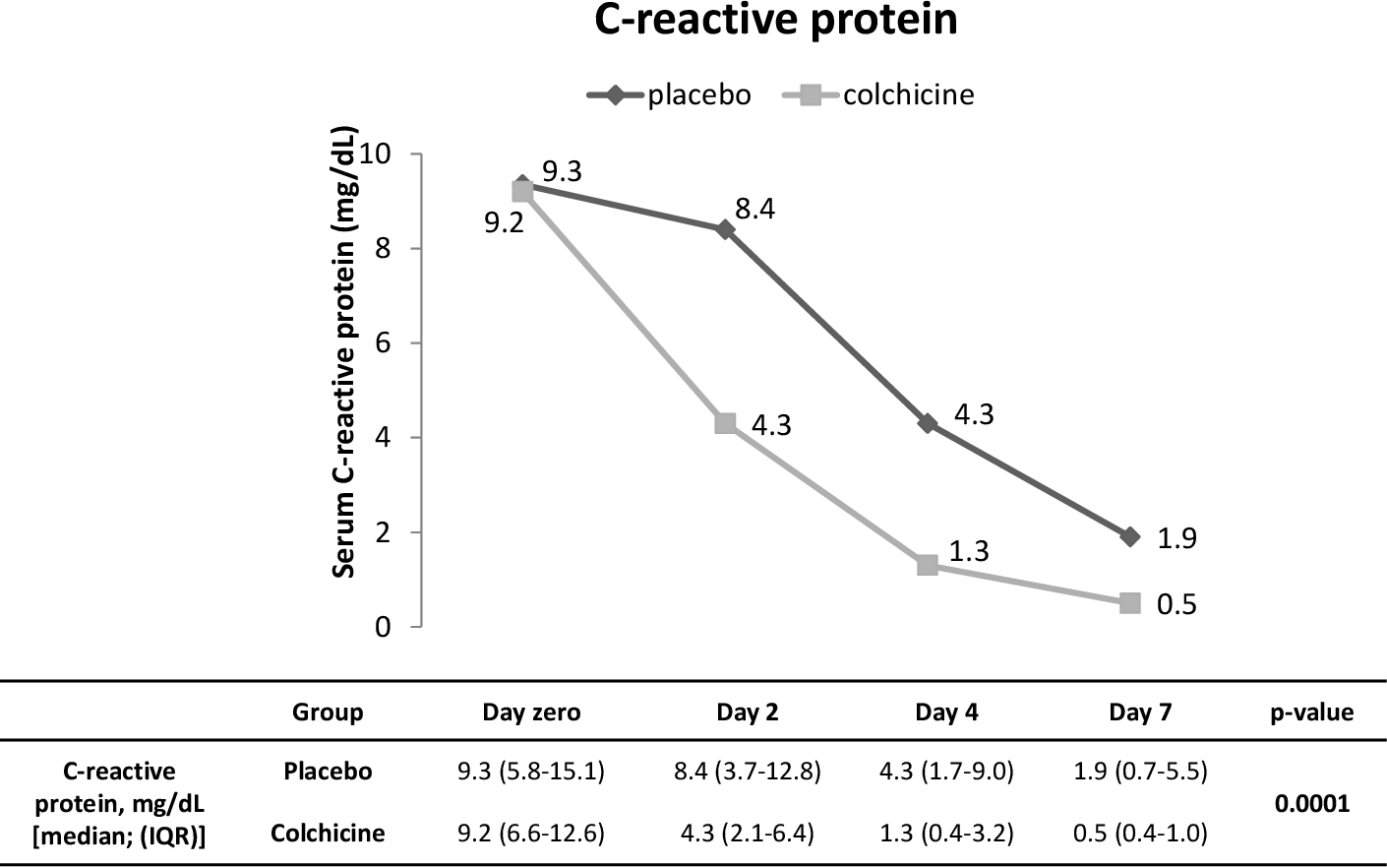
Temporal variation of serum C reactive protein from day 0 to day 7 for both groups.

We evaluated some laboratory parameters as secondary endpoints. Figure 4 and online supplemental figures 1 and 2 show the temporal variations of serum CRP and LDH and peripheral blood relation neutrophil to lymphocyte, respectively, from day 0 to day 7. Starting both groups at similar levels of serum CRP, at day 4, patients of the colchicine group presented significant reduction compared with themselves and with patients of the placebo group at day 0 (p<0.001). It is possible to observe that serum levels of CRP became different between the groups in the interval between days 2 and 4, with values near normal range (median=1.3 mg/dL) for the colchicine group at day 4. For the placebo group, statistical difference compared with the baseline occurred at day 7 (p<0.001), but no return to normal range of median CRP was observed at that time. The post-test for LDH showed a difference between day zero and days 4 and 7 for the colchicine group. No difference inter and intragroup for the relation neutrophil to lymphocyte was obtained.

10.1136/rmdopen-2020-001455.supp1 Supplementary data

10.1136/rmdopen-2020-001455.supp2 Supplementary data

The majority of adverse events (table 2) was mild (exception for pneumonia) and, to some extent, attributable to the viral infection itself or its complications, not entailing patients withdrawal. It seems to be the case of aspartate aminotransferase and alanine aminotransferase transient elevations, under 3× the upper limit of normal, with no difference between the groups (data not shown). New or worsened diarrhoea was more frequent in the intervention group (17% vs 6%). None of the patients suffered dehydration, and the diarrhoea was controlled with the prescription of an antisecretory agent (eg, racecadotril). Cardiac adverse events, undoubtedly the main issue on the use of hydroxychloroquine and/or azithromycin for COVID-19, did not have an augmented frequency by adding colchicine. No participant had QT interval above 450 ms during the observational period (data not shown). No difference between the groups on QT interval variation was observed from the value of day 0 to the highest value.

**Table 2:** Adverse events

Adverse event	Placebo group (n=36)	Colchicine group (n=36)	P value
AST transient elevation (n (%))	3 (8)	4 (11)	1
ALT transient elevation (n (%))	5 (14)	5 (14)	1
Nausea/vomiting (n (%))	4 (11)	2 (6)	0.67
Abdominal pain (n (%))	4 (11)	4 (11)	1
New or worsened diarrhoea (n (%))	2 (6)	6 (17)	0.26
Nosocomial pneumonia (n (%))	5 (14)	3 (8)	0.71
Arrythmia (n (%))	0	0	1
QT interval at day 3 (ms; median (IQR))	405 (388–406)	408 (392–415)	1
QT interval variation (ms; median (IQR))	28 (13–35)	22 (9–29)	0.25

ALT, alanine aminotransferase; AST, aspartate aminotransferase.

## Discussion

We presented the results of a randomised, double-blinded, placebo-controlled clinical trial of colchicine for COVID-19. Patients receiving colchicine abandoned oxygen supplementation earlier than those receiving placebo (median: 4.0 vs 6.5 days) and the time until discharge followed a similar tendency. These two primary endpoints have relevance for daily practice in the COVID-19 pandemic, by reducing the length of hospitalisation, consequently diminishing costs and the need for hospital beds. Besides that, the treatment with colchicine is not expensive.

Mainly obese women represented the study population (39 women and 33 men). Although the susceptibility to SARS-CoV-2 infection seems not related to gender, the clinical presentation of COVID-19 was proved worse in men, with higher rates of mortality.^19^ We also could not evaluate mortality rate by gender, but the two patients who died were men. The higher frequency of women in our study population comes from the fact that 19 out of 29 patients that met exclusion criteria were men with critical forms of the disease due to respiratory failure at the baseline evaluation.

A wide variation of the magnitude of systemic inflammation, reflected in the broad ranges of serum CRP (2.3–28.2 mg/dL) and SatO_2_/FiO_2_ (65–400), assures that this sample of patients is widely representative of the hospitalisations due to COVID-19 pneumonia. Taking into account the effect on both, the CRP curve and the need for supplemental oxygen, colchicine use seems promising if we consider that the systemic inflammation was safely halted in a shorter period compared with the standard treatment.

Six patients needed ICU admission and all of them had SatO_2_/FiO_2_ <100 at enrolment. It is certain that the effect of colchicine in reducing systemic inflammation is not enough for every patient and that for some of them no intervention would prevent respiratory failure. These six patients received methylprednisolone. Worthy of mention, the concomitant use of a glucocorticoid should not be considered to have influence on the more favourable evolution of the intervention group, once both groups had similar number of randomised patients receiving the drug.

The study protocol used the maximum safe daily dose of colchicine^20^ considering a body weight of 50 kg, that is, 0.030 mg/kg. Body weight of the participants ranged from 62 to 145 kg, which results, for some patients, in a daily dose under 0.015 mg/kg, the minimal dose for chronic use of colchicine. This range was maintained, even for the first 24 hours, aiming to avoid the occurrence or worsening of diarrhoea, a frequent manifestation of COVID-19.

Only 5 out of 72 analysed patients had normal values of BMI, four of them randomised to the placebo group. Systemic inflammation is a common characteristic linking obesity, metabolic syndrome and elevated cardiovascular risk,^21 22^ with highlighted role for IL-1β and NLRP3 inflammasome. The adipose tissue of obese has macrophage type 1 as the main resident phagocytic cell. This subgroup of macrophages is primed to secrete proinflammatory cytokines, such as IL-1β, IL-6 and TNF.^23^ Some groups found obesity a risk factor for severity of COVID-19, as reported in a systematic review with meta-analysis.^24^ Because of the small number of non-obese in our study population, we cannot simply extrapolate our findings to non-obese individuals of the general population, once the action of colchicine may be limited to those in which the association of obesity and inflammation has a pivotal pathophysiological effect.

Colchicine was already tested as protective against ischaemic events postmyocardial infarction with some success,^25^ and it was found to ameliorate endothelial function in a subgroup of patients with coronary disease and augmented peripheral blood leucocyte count.^26^

Recently, our group reported two possible mechanisms involved on lung inflammation in COVID-19. First, we reported the possible role for neutrophil extracellular traps on lung inflammation in COVID-19.^6^ Second, we found that the activation of NLRP3 inflammasome was associated with the severity of COVID-19 and poor clinical outcome.^27^ One of the actions of colchicine is to reduce migration of leucocytes, mainly neutrophils, to inflamed tissues.^9^ Whatever the mechanism of action—inhibiting inflammasome, reducing neutrophil migration and activation or preventing endothelial damage–colchicine seems to be beneficial for the treatment of hospitalised patients with COVID-19. The markedly reduction of serum CRP levels between second and fourth days coincides with clinical recovery of the majority of patients. Most of the participants were in the second week of symptoms, a phase in which systemic inflammation becomes striking. It is very unlikely that colchicine has some antiviral effect.

Worthy of note is the fact that the drug did not contribute to hepatic or cardiac adverse events nor caused immunosuppression. Diarrhoea was not a limiting adverse event, and an antisecretory drug may be added when necessary.

The GReek Study in the Effects of Colchicine in Covid-19 Complications Prevention (GRECCO-19), an open-label randomised clinical trial, had some results recently published.^15^ The authors found that patients receiving colchicine were less prone to clinical deterioration, despite the fact that their serum levels of CRP showed no significant difference compared with those of patients not receiving the drug. For a matter of comparison, when analysing patients with body weight >60 kg in use of azithromycin (approximately 92%), patients of GRECCO-19 study received a total dose of 5.0 mg of colchicine in the first 5 days, while in our study, patients received 7.5 or 8.0 mg in the same period. This difference of dosage of ≥50% for the first 5 days may explain the evident reduction of serum CRP in the present study and justify the better evolution of the intervention group. Scarsi *et al*^14^ showed an association between treatment with colchicine and improved survival in a single-centre cohort of adult hospitalised patients with COVID-19 pneumonia and SARS. They conclude that their results may support the rationale of colchicine use for the treatment of COVID-19 and that the efficacy and safety must be determined in controlled clinical trials. Authors used the dose of 1.0 mg/day for all patients with reduction to 0.5 mg if diarrhoea occurred.

Our clinical trial has some limitations. The most evident is the reduced number of patients and the impossibility to evaluate the capacity of colchicine to avoid admission to ICU and reduce mortality. To balance this, the blinding and the control by a placebo arm strengthen our finds. The absence of mechanistic investigations, for example, measures of the plasmatic levels of cytokines, is another limitation. Our exclusion criteria were some kind of restrictive, such as the prohibition of some cardiovascular drugs. Much of this concern was related to drugs that could impair colchicine metabolism or excretion, but some concern was also due to the potential hazardous effect of hydroxychloroquine and azithromycin combined use for myocardial fibres. Finally, as patients were discharged, the number of blood samples reduced through the first week of observation and beyond, once it would not be appropriate to summon up the patients for new blood collections due to SARS-CoV-2 transmission possibility.

## Conclusions

Patients who received colchicine in this randomised, double-blinded, placebo-controlled clinical trial presented better evolution in terms of the need for supplemental oxygen and the length of hospitalisation. Serum CRP was a laboratory marker of clinical improvement. Colchicine was safe and well tolerated. Clinical trials with larger numbers of patients should be conducted to further evaluate the efficacy and safety of colchicine as an adjunctive therapy for hospitalised patients with moderate to severe COVID-19.
