# Supplementary material for: Beneficial effects of colchicine for moderate to severe COVID-19: a randomised, double-blinded, placebo-controlled clinical trial
Source: RMD Open. 2021 Feb 4;7(1):e001455. doi: 10.1136/rmdopen-2020-001455 (PMC7868202; doi:10.1136/rmdopen-2020-001455)
Supplement: Supplementary data [file rmdopen-2020-001455supp001.pdf]

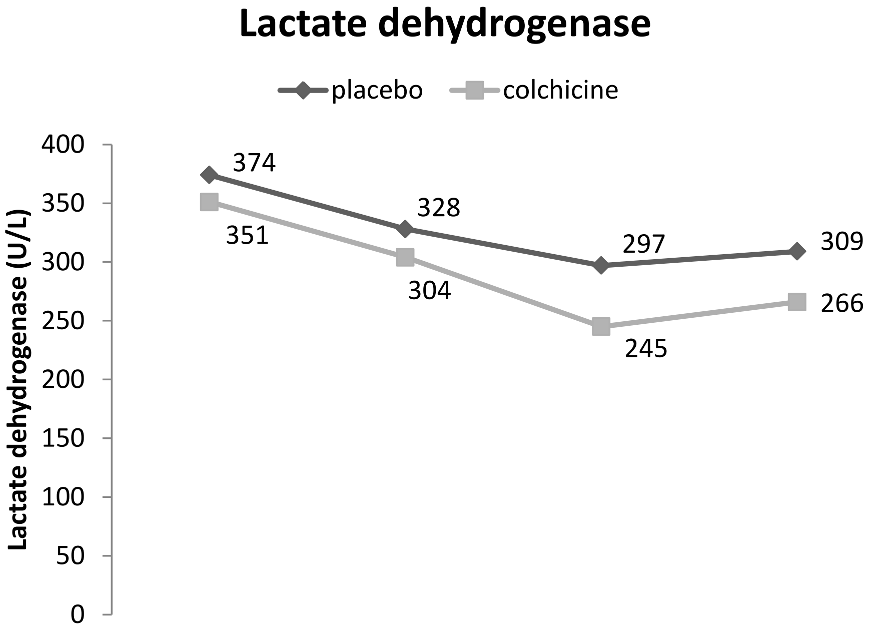

	Group	Day zero	Day 2	Day 4	Day 7	p-value
**Lactate dehydrogenase U/L [median; (IQR)]**	**Placebo**	374 (301-477)	328 (270-430)	297 (248-371)	309 (227-385)	**<0.001**
	**Colchicine**	351 (299-473)	304 (250-390)	245 (201-290)	266 (209-315)	

IQR – Interquartile range

Supplemental Figure 1. Temporal variation of Lactate dehydrogenase from D zero to D7 for both groups
