# Supplementary material for: Beneficial effects of colchicine for moderate to severe COVID-19: a randomised, double-blinded, placebo-controlled clinical trial
Source: RMD Open. 2021 Feb 4;7(1):e001455. doi: 10.1136/rmdopen-2020-001455 (PMC7868202; doi:10.1136/rmdopen-2020-001455)
Supplement: Supplementary data [file rmdopen-2020-001455supp002.pdf]

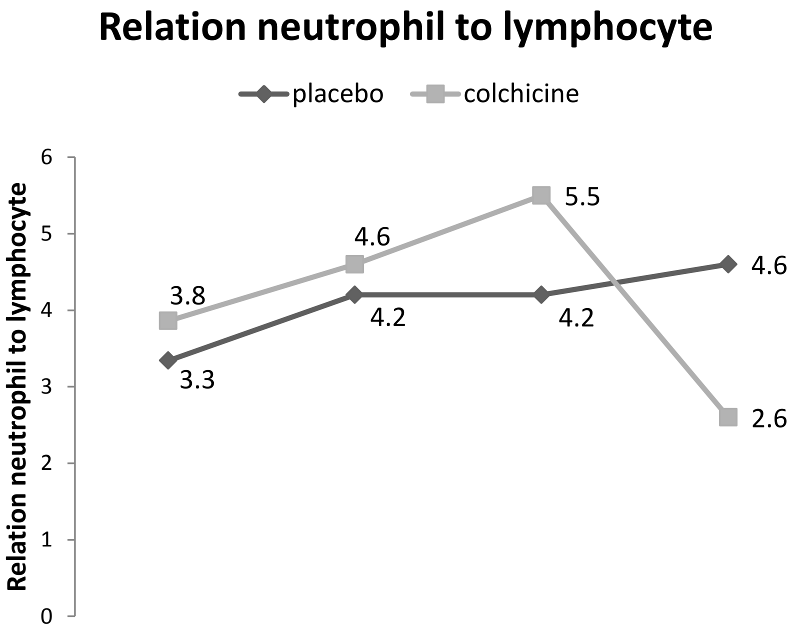

	Group	Day zero	Day 2	Day 4	Day 7	p-value
**Neutrophil to lymphocyte [median; (IQR)]**	**Placebo**	3.4 (2.2-6.2)	4.1 (2.8-7.6)	4.0 (2.6-8.1)	3.5 (1.6-8.2)	0.55
	**Colchicine**	2.8 (2.4-4.4)	3.7 (2.1-6.7)	3.0 (2.0-7.7)	1.5 (1.3-5.6)	

IQR – Interquartile range

Supplemental Figure 2. Temporal variation blood relation neutrophil to lymphocyte from D zero to D7 for both groups
